# Impact of Implementing a Standard Operating Procedure to Reduce Blood Wastage in Blood Centers of Iran

**DOI:** 10.34172/aim.2024.14

**Published:** 2024-02-01

**Authors:** Hayedeh Javadzadeh Shahshahani, Shahin Sharifi, Soheila Nasizadeh

**Affiliations:** ^1^Blood Transfusion Research Center, High Institute for Research and Education in Transfusion Medicine, Tehran, Iran

**Keywords:** Blood component, Blood transfusion, Implementation, Plasma, Platelet, Waste

## Abstract

**Background:**

Blood wastage leads to additional costs and reduced blood availability to patients. Above all is the moral issue of wasting donor gifts. This study aimed to determine the rate of blood wastage before and after implementing a new standard operating procedure (SOP) in Iran.

**Methods:**

In this interventional study, a SOP for wastage management was prepared and implemented in all blood centers throughout the country. Data were extracted from the integrated software of the Iranian Blood Transfusion Organization (IBTO). The wastage rate of blood components in the post-intervention years (2016-2017) was then compared with that in the pre-intervention years (2013-2015) using the Z test.

**Results:**

The overall wastage rate decreased by 36.86% (*P*<0.001, 95% CI [36.84-36.88]) after the intervention. Red blood cell (RBC) wastage decreased from 2.6% to 2.5%, platelet wastage from 19.5% to 10.6% and plasma wastage from 15.5% to 7.3% (*P*<0.001). The highest percentage of waste reduction pertained to plasma components, which decreased by 52.90% (*P*<0.001, 95% CI [52.86-52.94]). Expiration was the most common cause of RBC and platelet wastage. The most common causes of plasma wastage were RBC contamination and rupture or leakage of the bags. The intervention resulted in a drop of over 250000 discarded components each year, equal to approximately thirty-six million dollars in savings.

**Conclusion:**

This intervention effectively reduced waste and increased efficiency. Ongoing blood wastage reviews, auditing, and receiving feedback from the central headquarters were powerful tools in following the compliance of blood centers. Further studies are recommended, especially concerning blood wastage in hospital blood banks and various wards.

## Introduction

 Blood components are valuable resources and wastage should be avoided to protect the interests of all blood donors and potential recipients. Wastage of blood components should be considered in the context of several issues: ethical issues and saving the blood components. Blood is only available through altruistic donations by voluntary non-remunerated blood donors; therefore, every effort must be made to reduce wastage. The dedication and time it takes to donate voluntarily and altruistically must be taken seriously, and the fact that blood donors hope that their donations will benefit a patient is of utmost importance. Therefore, every effort must be made to reduce the number of wasted blood units. Wastage of blood components has a significant impact on the financial resources of healthcare systems and adequacy of the blood supply. Reducing blood wastage has the potential cost-saving effect by increasing blood components available for specific patients’ needs.

 The wastage rate in different studies varies from 0.2 to 30%.^1^ In Brazil, the discard rate of blood components ranges from 10% to 20%. Various studies have used different strategies to reduce blood component discard rate.^2-5^ However, no comprehensive research on blood waste management has been previously conducted in Iran. In 2011-2012, efforts were focused on increasing the number of blood donations, which ultimately lead to an increase in wasted blood units. The Yazd Blood Center performed a pilot study in 2013 in which an intervention was applied; the results showed that blood wastage was reduced by 60%.^6^ Then, this was conducted in blood centers across the country as an interventional study aiming to investigate the impact of the implementation of the standard operating procedure (SOP) for blood wastage management.

## Materials and Methods

 This is a before and after interventional study. Initially, a SOP was prepared (TM.064.WOI) for blood waste management in blood centers and implemented from the beginning of 2016. In this SOP, the most common conditions that could cause blood wastage were described as blood components that did not fulfill the standards of medical centers or fractionation companies for use during collection, preparation and storage. The leading causes for waste included expiration, inadequate volume, hemolysis of red blood cell (RBC) units, contamination of plasma or platelets with RBCs, blood container leakage, reactive ELISA test results and improper temperature during storing or transport. Then, the following measures were proposed to reduce the waste.

 The optimal inventory level for RBC units was re-evaluated in order to decrease blood wastage due to expiration. Previously ER.002.WOI stated that the inventory level for RBC units should be 7 days of hospitals’ needs; a recent update reduced it to 5 days of hospitals’ needs. Surplus blood was sent to other blood centers in need. It was recommended that blood centers limit blood donor recruitment and cancel mobile teams when other centers do not need additional units. Strict adherence to previous SOPs on managing blood center inventory and issuing blood components to medical centers was also recommended to reduce outdated blood units.

 Maintaining the proper temperature during transport and storage is essential to avoid bacterial contamination and RBC hemolysis, thus avoiding blood wastage. Providing electronic temperature monitoring as well as timely maintenance, calibration, and validation of storage equipment were emphasized. A backup cooling system was also recommended for all blood centers as a substitute for the storage of blood components when the function of their refrigerators and/or freezers failed.

 Timely maintenance, calibration, and validation of centrifuges are of utmost importance in obtaining platelets and plasma with minimal RBC contamination. Thus, this was also emphasized in order to minimize waste.

 Continual training courses were presented to advance the performance of staff members to minimize technical errors leading to the wastage of blood components. These educational programs included standard methods of blood collection, processing, storage, and transportation according to the Standard Operational Procedures of the Iranian Blood Transfusion Organization (IBTO). These SOPs are an essential part of the integrated quality assurance system throughout the blood centers of Iran to ensure the quality of blood components. Regular internal and external audits and preventive and corrective actions are performed to improve the effectiveness of the quality system. Monitoring of blood component wastage, reporting results of waste monitoring on a monthly basis to the Technical Department at central headquarters, and reviewing feedback received from the headquarters at regular intervals were also required.

 The impact of these interventions was evaluated from the beginning of 2016 to the end of 2017. The outcome, including the reduction of blood component wastage, was determined for all 31 provincial blood centers in Iran. A unique number (1 to 31) was assigned to each provincial blood center. In addition to the data pertaining to the number and types of blood components prepared in blood centers of Iran, data was also collected regarding the number and types of blood components wasted. These data were extracted from the integrated software of the IBTO and documented in the forms specified in 00.TM.293.FRM. The forms were regularly investigated by the headquarter. Any missing or incomplete data were sent back to be completed based on data in the software. Blood components requested from hospital blood banks and issued to them also exist in our computer data. Data was collected annually from 2013 up to the end of 2017 in each blood center. The ratio of blood component waste was calculated by the number of RBC, platelets, and plasma components (including fresh frozen plasma and cryoprecipitate poor plasma) wasted divided by the number of same blood components prepared each year. In other words, only one ratio was calculated for each type of blood component annually in each blood center. In addition, the ratio of total waste was calculated by dividing the wastage of total blood components by the total number of blood components prepared. The total wastage ratio of the country was calculated as a percentage of the total number of blood components prepared in the country. The results obtained from the years before the intervention (from 2013 to the end of 2015) were compared with post-interventional data (from 2016 to the end of 2017). They were analyzed using descriptive statistics and the test of two proportions (Z test).

## Results

**Figure 1:**
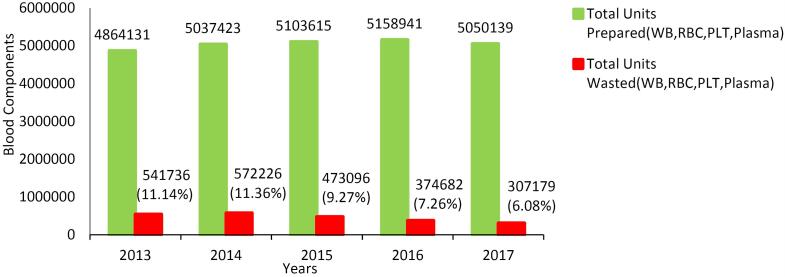
Total Number of Blood Components Prepared (Whole Blood, RBC, Platelet and Plasma) and Those Wasted From 2013 to 2017 in All Blood Centers of Iran

 The total number of blood components prepared as well as those wasted in Iran from 2013 to the end of 2017 is shown in Figure 1. The frequency of blood components prepared and wasted throughout the country in the years before and after the intervention is shown in Table 1. After the intervention was implemented, the wastage rate decreased by 36.86% (*P*< 0.001, 95% CI [36.84- 36.88]).

**Table 1:** Comparison of the Frequency of Blood Components Prepared and Wasted in the Years Before and After the Intervention in Overall Blood Centers of Iran

**Phase**	**Blood Components** **(Whole Blood, RBC, Platelet, and Plasma)**		
	**Prepared** **N**	**Wasted** **N **	Wastage ratio (%)^*^ **(95% CI)**
Pre-intervention (2013–2015)	15005169	1587058	10.58 (10.56-10.59)
Post-intervention (2016–2017)	10209080	681861	6.68 (6.66-6.69)

^*^Wastage ratio calculated as a percentage of the number of units prepared.

**Figure 2:**
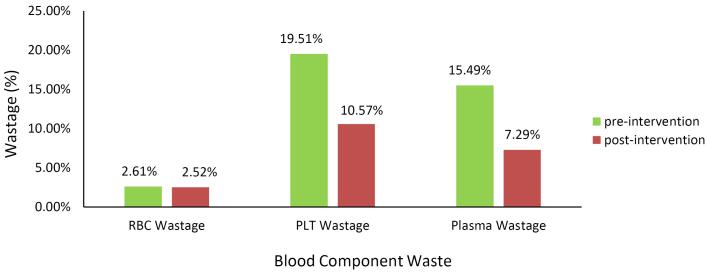
Percentage of Each Blood Component Wasted Before and After the Intervention in Iran

Figure 2 shows the percentage of each blood component wasted before and after the intervention. This figure shows that the highest rate of waste was related to platelets, followed by plasma; the lowest rate was related to RBC units. The highest percentage of waste reduction was related to plasma units with 52.90% (*P* < 0.001, 95% CI [52.86-52.94]). The platelet wastage rate decreased by 45.82% (*P* < 0.001, 95% CI [45.76 - 45.88]). RBC waste showed a decrease by 3.44% (*P* < 0.001 CI [3.43 - 3.45]).

 It is noteworthy that in the pre-intervention years, dispatching surplus blood components to other blood centers with lower inventory levels amounted to 436 282 units, 7.09% of the total blood units collected (6 154 265 units). After the intervention, the dispatch of surplus blood increased to 8.0% of the total blood units collected (337 802 units out of 4 220 610 units) (*P* < 0.001).

###  The 28 Provincial Blood Centers

**Figure 3:**
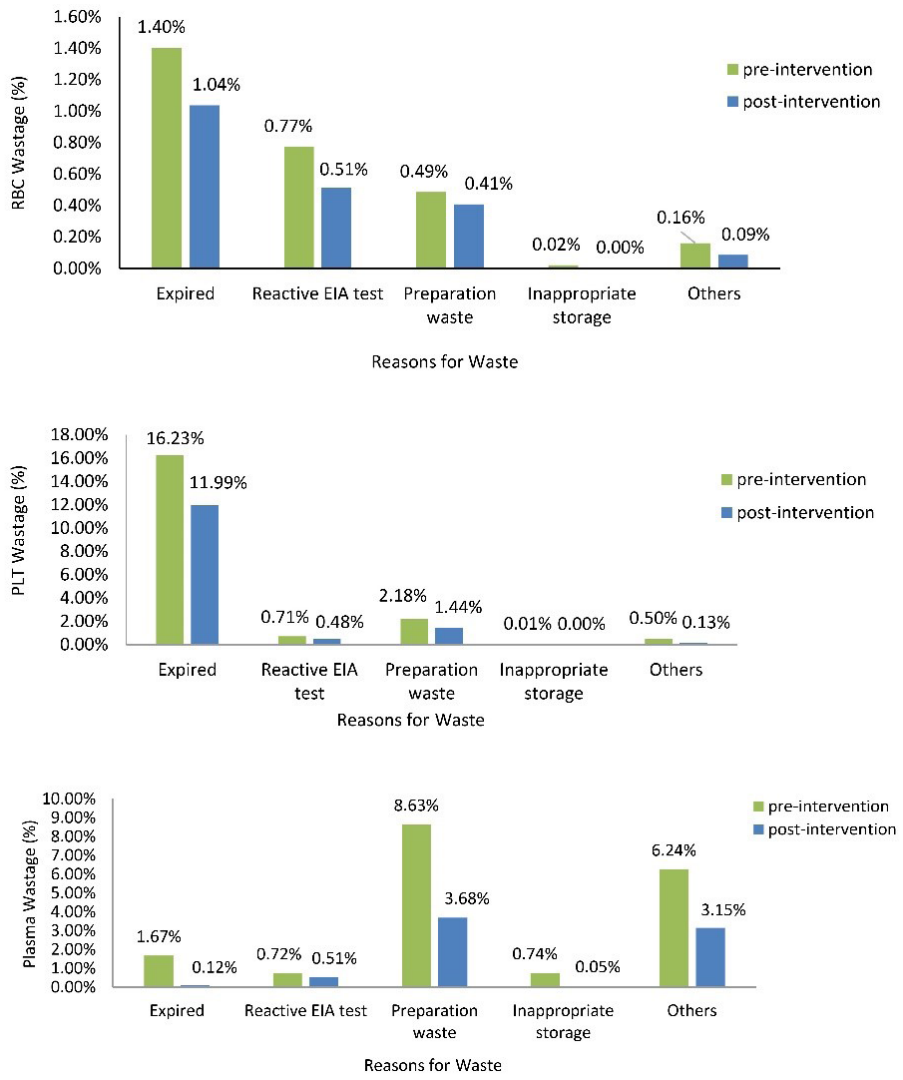
Frequency of Different Reasons for RBC, Platelet, and Plasma Wastage in Pre-intervention Years Compared to Post-intervention Years in 28 Provincial Blood Centers

 In 28 out of 31 provincial blood centers, the wastage of all three types of blood components was reduced after the implementation of this intervention (*P* < 0.001); the total waste of the blood components decreased from 12.6% to 6.6% (47% reduction) (*P* < 0.001, 95% CI [46.97-47.03]). Figure 3 shows the frequency of different reasons for RBC, platelet, and plasma wastage before and after the intervention in these 28 provincial blood centers. The frequency of different reasons for RBC, platelet and plasma wastage in “all blood centers of Iran” decreased, which was in agreement with 28 blood centers. The most frequent reason for RBC wastage was expiration, followed by ELISA reactive test results. The major cause of platelet wastage was platelet expiration; technical fault during preparation was also a common cause. The most common cause of plasma wastage was related to plasma preparation, which was due to factors such as improper volume, RBC contamination, and rupture or leakage of the plasma bags. In these blood centers, the number of blood components issued to hospitals and surplus RBCs dispatched to other blood centers increased after implementing this intervention (*P* < 0.001; Table 2).

**Table 2:** Frequency of Blood Component Wastage, Issued to Hospitals and Surplus Dispatched to Other Centers in 28 Provincial Blood Centers Before and After the Intervention

**Number of **	**Pre-intervention (2013-2015)**	**Post-intervention (2016-2017)**
Blood components prepared	10589382	7153807
Blood components wasted (%)^a^	1334120 (12.60%)	472902 (6.61%)
Blood components requested by hospitals	7549653	5278721
Blood components issued to the hospitals (%)^b^	6704092 (88.80%)	4818173 (91.30%)
Blood units collected	3528689	2091319
Surplus RBC units dispatched to other blood centers (%)^c^	271557 (7.7%)	166369 (8.0%)

^a^Wasted blood components calculated as a percentage of the number of blood components prepared. ^b^Issued blood components calculated as a percentage of blood components requested by hospitals. ^c^Dispatched RBC units calculated as a percentage of collected blood units.

###  The Remaining 3 Provincial Blood Centers

 In the remaining three blood centers (#16, #19 and #26), RBC wastage increased in the years following the implementation of this intervention (*P* < 0.001). The main cause of RBC wastage in these blood centers was expiration.

**Figure 4:**
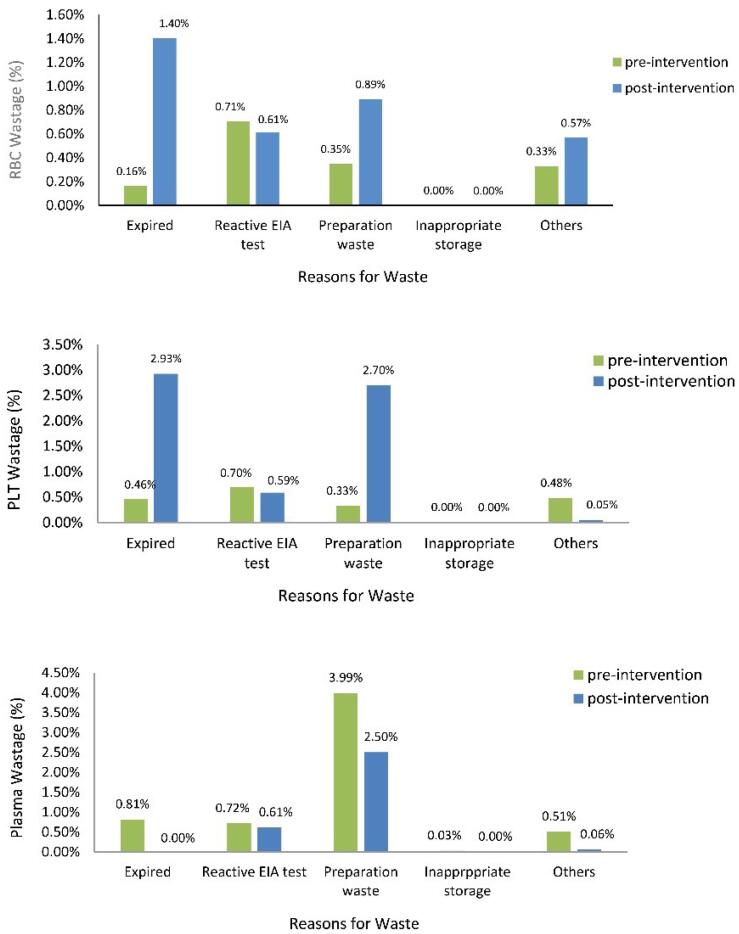
Frequency of Different Reasons for RBC, Platelet, and Plasma Wastage Before and After the Intervention in Blood Center #16

 In Blood Center #16, RBC wastage increased from 1.6% to 3.5% and platelet wastage also increased from 3.0% to 6.3%. The most common causes of RBC and platelet wastage were expiration and inappropriate preparation of the blood components (*P* < 0.001) (Figure 4). Inappropriate platelet production included cases of RBC contamination and inadequate volume. However, in the other two centers, a decrease in platelet wastage was seen. Platelet wastage decreased from 12% to 7% in Blood Center #19 and from 7% to 3% in Blood Center #26 (*P* < 0.001). Plasma wastage, however, decreased in all three of these blood centers.

## Discussion

 The present study was the first national interventional study aiming to reduce the amount of blood wastage in Iranian blood centers, which followed a pilot study at the Yazd Blood Center.^6^ In 2013, a total of 4 864 131 blood components were prepared in Iran, of which 11% (541 736 units) were wasted. After the SOP was implemented, the wastage rate was reduced to 6% in 2017 (Figure 1). In Australia, a national improvement strategy for blood products managed to decrease wastage from 7.3% in 2013 to 5.4% in 2017.^7^ The ultimate goal in wastage reduction is to ensure that blood components are available when they are needed. No matter what strategy is chosen, a certain level of blood component wastage is inevitable. Nonetheless, there are areas where blood component wastage is avoidable and every effort should be made to reduce the number of wasted blood units. This study focused on reducing the proportion of wasted blood components.

 Platelets had the highest wastage rate among the blood components prepared before as well as after implementing the intervention (Figure 2). Outdate rates were reported to be more than 10 to 20% worldwide.^8^ In the United States and Western Europe, wastage rate was reported to be 15 to 20%.^9^ In the Yazd study, the platelet wastage rate was 18.5%.^6^ In this study, in 6 of the blood centers, over 60% of the prepared platelet units were discarded before the intervention. Blood Center #29 had the highest percentage of reduction in platelet wastage (79%), decreasing from 24% to 5% after implementing the intervention. In Blood Center #16, platelet outdating increased after the intervention, although its platelet wastage rate was lower than the national average wastage rate. This increase in outdating could have been due to an increase in platelet preparation in order to ensure an adequate supply to meet the hospitals’ needs. The short shelf-life of platelet units and unpredictable hospital demands are all reasons that can lead to an increase in discarding this blood component. In addition, the transfer of excess platelets to other provincial blood centers is limited by platelet storage temperature and its short life. To reduce the number of expired platelet units, preparing platelets on a weekly basis can help reduce platelet wastage. Schilling et al showed that statistical and deep neural network models allow for predictions of platelet demand with sufficient accuracy to significantly reduce waste and shortage.^10^ Other factors that resulted in platelet wastage were RBC contamination and improper volume. The use of up-to-date centrifuges could result in a decrease in platelet wastages due to these factors.

 In this study, out of 5 983 430 RBC units prepared in the years before the intervention, 155 925 RBC units (2.6%) were discarded. In pre-intervention years, the highest percentage of RBC wastage occurred in three blood centers and was approximately 10%. In a study in India, 6.6% of whole blood or RBC units were discarded. Expiration was the most common cause of discarding, leading to a loss of US$ 100 000.^11^ After the intervention, the highest percentage of reduction in RBCs wastage was related to Blood Center #30 at 80%. In this Blood Center, RBC wastage decreased from 8.9% to 1.3% and the expiration rate decreased significantly (from 7% to 0.6%). Results from the Yazd study showed that, in the pre-intervention years, RBC wastage was 10%, which reached 3% in the post-intervention years.^6^ In Australia, a strategy to reduce RBC wastage involved reducing the level of national RBC inventory and increasing the efficiency of the blood collection system. By implementing the national wastage reduction strategy from 2013 to 2017, RBC national discards decreased from 5.0% to 2.3%.^7^ Similarly, in the present study,the decrease in RBC inventory level reduced the expired RBC units.On the other hand, RBC wastage increased in Blood Centers #16, #19, and #26 after the intervention. These blood centers received blood from other blood centers and this could have caused an increase in RBC wastage. In some cases, the blood units received from other blood centers had blood groups that were not needed or did not meet the cold room storage requirements. Therefore, coordination between the distribution authorities of the receiving and sending blood centers should be improved to prevent blood wastage. In addition, inventory management in blood centers is complex and challenging. Studies have shown that machine learning or simulation models for inventory management could decrease the wastage of RBCs in blood centers.^2-4,12^

 The results of this study showed that out of 5 979 484 plasma units prepared in the years before the intervention, 926 073 (15.5%) were wasted. Plasma wastage during this period was encountered predominantly in five blood centers, with more than 40% of their plasma units being wasted. In Blood Center #31, nearly 60% of the plasma units were discarded. After the intervention, plasma wastage in Iran reached 7% mainly due to a decrease in problems encountered during the preparation stage such as RBC contamination and rupture or leakage of plasma bags. Expiration and broken bags were the main cause of plasma wastage in other studies.^13^ Blood Center #6 and #30 had the highest percentage of reduction (90%) in plasma wastage (from 37% to 4% and from 42% to 5%, respectively). In Blood Center #6, outdated units were the main reason for wastage before the intervention, which decreased significantly from 30% to 0.3%.In the majority of blood centers of Iran, plasma outdating decreased by sending surplus plasma for fractionation. In the Yazd study, plasma expiration decreased from 20% to zero. Plasma shipments for fractionation increased from 50% to more than 70%.^6^ In Blood Center #30, plasma wastage was mainly due to inappropriate storage conditions (24%), which dropped to 2% following the use of calibrated and validated equipment. They were equipped with an electronic temperature monitoring system and alarm system with audible alarms along with text alerts (SMS) to key personnel. All transport equipment, packaging materials, and methods were validated for different transport times and ambient temperatures. Studies have shown that using updated and validated cooling systems could significantly decrease plasma and RBC wastage.^14,15^ The use of Thawed plasma and extended shelf-life of plasma is increasingly rising in some countries as a strategy to reduce plasma wastage and increase plasma availability in traumas and many other clinical situations.^16,17^

 During this study, only 0.5% of blood components were discarded due to reactive test results for blood-borne infectious diseases. This portion of discarded units was inevitable. In some studies, the main reason for blood wastage was positive serological markers.^18,19^ In the study by Monich et al, the discard rate due to positive serology decreased from 10% to 4.9%. Educational programs for the general population and vaccination for hepatitis B were efficient actions in discard reduction. In another study in Brazil, 3.33% of donations were discarded due to positive serological markers, which exceeded the rate in developed countries.^18^

 After implementing the wastage management SOP, for each one million RBC, plasma, and platelet units produced, 900, 83 000, and 90 000 fewer units have been wasted, respectively. A total of twenty-four million dollars was saved for the production of one million units of each type of blood component, according to a study by Davoudi-Kiakalayeh et al on the cost of RBC, plasma, and platelet units.^20^ The ratio of blood components issued to medical centers and the percentage of blood units transferred to other blood centers have also improved (*P* < 0.0001; Table 2).

 Providing blood centers with statistical data regarding their own wastage rates, as well as data from other centers also had a powerful impact in increasing efficiency. Timely regular reports from the central headquarters helped maintain awareness and interest in identifying strategies for reducing avoidable wastage.

## Conclusion

 The intervention applied in this study was effectively associated with wastage reduction in blood centers throughout Iran. Regular auditing and monitoring of wasted blood components and performing corrective actions in cases of non-conformity were effective measures. With respect to the problem encountered in three of the blood centers that lead to an increase in RBC wastage, efforts should be made to improve collaboration between blood centers involved in the transfer of surplus blood components and ultimately to reduce blood wasted in this process. Establishing a software connection between blood centers and hospital blood banks could help further improvement of blood inventory management. Further studies are recommended, especially concerning blood wastage in hospital blood banks and various wards.
